# Comparison of Military Health System Data Repository and American College of Surgeons National Surgical Quality Improvement Program-Pediatric

**DOI:** 10.1186/s12887-019-1795-x

**Published:** 2019-11-08

**Authors:** Arin L. Madenci, Cathaleen K. Madsen, Nicollette K. Kwon, Lindsey L. Wolf, Kristin A. Sonderman, Jill M. Zalieckas, Samuel E. Rice-Townsend, Adil H. Haider, Robert L. Ricca, Brent R. Weil, Christopher B. Weldon, Tracey P. Koehlmoos

**Affiliations:** 1 Department of Surgery, Boston Children’s Hospital and Harvard Medical School, Boston, MA USA; 2 Department of Surgery, Brigham and Women’s Hospital and Harvard Medical School, Boston, MA USA; 3 Center for Surgery and Public Health, Boston, MA USA; 4 Henry M. Jackson Foundation for the Advancement of Military Medicine, Inc., Bethesda, MD USA; 5 Uniformed Services University of the Health Sciences, Bethesda, MD USA

**Keywords:** Health services research, Pediatrics, General surgery

## Abstract

**Background:**

Given the rarity of pediatric surgical disease, it is important to consider available large-scale data resources as a means to better study and understand relevant disease-processes and their treatments. The Military Health System Data Repository (MDR) includes claims-based information for > 3 million pediatric patients who are dependents of members and retirees of the United States Armed Services, but has not been externally validated. We hypothesized that demographics and selected outcome metrics would be similar between MDR and the previously validated American College of Surgeons National Surgical Quality Improvement Program-Pediatric (NSQIP-P) for several common pediatric surgical operations.

**Methods:**

We selected five commonly performed pediatric surgical operations: appendectomy, pyeloplasty, pyloromyotomy, spinal arthrodesis for scoliosis, and facial reconstruction for cleft palate. Among children who underwent these operations, we compared demographics (age, sex, and race) and clinical outcomes (length of hospital stay [LOS] and mortality) in the MDR and NSQIP-P, including all available overlapping years (2012–2014).

**Results:**

Age, sex, and race were generally similar between the NSQIP-P and MDR. Specifically, these demographics were generally similar between the resources for appendectomy (NSQIP-P, *n* = 20,602 vs. MDR, *n* = 4363; median age 11 vs. 12 years; female 40% vs. 41%; white 75% vs. 84%), pyeloplasty (NSQIP-P, *n* = 786 vs. MDR, *n* = 112; median age 0.9 vs. 2 years; female 28% vs. 28%; white 71% vs. 80%), pyloromyotomy, (NSQIP-P, *n* = 3827 vs. MDR, *n* = 227; median age 34 vs. < 1 year, female 17% vs. 16%; white 76% vs. 89%), scoliosis surgery (NSQIP-P, *n* = 5743 vs. MDR, *n* = 95; median age 14.2 vs. 14 years; female 75% vs. 67%; white 72% vs. 75%), and cleft lip/palate repair (NSQIP-P, *n* = 6202 vs. MDR, *n* = 749; median age, 1 vs. 1 year; female 42% vs. 45%; white 69% vs. 84%). Length of stay and 30-day mortality were similar between resources. LOS and 30-day mortality were also similar between datasets.

**Conclusion:**

For the selected common pediatric surgical operations, patients included in the MDR were comparable to those included in the validated NSQIP-P. The MDR may comprise a valuable clinical outcomes research resource, especially for studying infrequent diseases with follow-up beyond the 30-day peri-operative period.

## Background

Analysis of large medical databases holds substantial promise for improving healthcare delivery, including potential contributions to predictive modeling, surveillance, and other health improvement initiatives [1]. Likewise, national databases aggregate factors that otherwise might be too rare for meaningful analysis. However, the usefulness of such databases depends on generalizability and suitability as a representative sample [1], which can be evaluated in part by comparison against an existing validated resource.

One such validated database for pediatric surgical research is the National Surgical Quality Improvement Program-Pediatric (NSQIP-P) database, overseen by the American College of Surgeons in conjunction with the American Pediatric Surgery Association. This database collects 94 data points on surgical patients under 18 years of age, with additional data points for neonates under 30 days of age, and follows patient outcomes for 30 days post-operatively [2].

Also potentially useful for pediatric surgical research is the claims database maintained by the United States Military Health System (MHS) known as the MHS Data Repository (MDR). The MHS includes a universally-insured population of 9.4 million military and civilian beneficiaries [3] including 3 million children and has been called “America’s ‘undiscovered’ laboratory for health services research” [4]. The beneficiary population is considered to be demographically representative of the adult U.S. population from age 18–64 years [5–7], however, to date, the pediatric generalizability has not been evaluated. Therefore, the aim of this research is to compare demographics and select outcomes (mortality, length of stay, and readmission) of the MHS Data Repository (MDR) to a validated resource, the NSQIP-P database, for five common pediatric surgical procedures to highlight the relative advantages and disadvantages of each resource. Findings of this research will help to evaluate the utility of the MDR as a tool for population-level research in pediatric procedures.

## Methods

### Study population

This study included data from two sources: 1) the United States Department of Defense Military Health System Data Repository (MDR) and 2) the American College of Surgeons Pediatric National Surgical Quality Improvement Project (NSQIP-P). The MDR is a claims database with records of healthcare delivered between 2005 and 2014 to over 3 million children who are dependents of active-duty personnel, retired service members, activated members of the National Guard and Reserve, civilian dependents of included personnel, and survivors or others entitled to care from the Department of Defense [4]. The MHS is separate from both the care provided for soldiers in combat zones and the Veterans Health Administration. Follow-up time for children, included as military-based insurance eligible dependents in the MDR, extends until their parent(s) or guardian(s) leave active duty without retiring or until 18 years of age or college graduation. A summary of other defining characteristics of the MDR is available in previously published work [8].

The NSQIP-P is a prospective clinical database which includes data from 54 participating hospitals in North America, which are abstracted by trained surgical clinical reviewers [9]. Patients under the age of 18 years who underwent selected general, neurosurgical, urological, otolaryngologic, plastic, and orthopedic procedures are eligible for selection by systematic sampling on an 8-day cycle. Only overlapping years, specifically 2012 to 2014, were included from both data sources.

We included five commonly performed operations across multiple disciplines that were captured by both the MDR and the NSQIP-P (2012–2014): appendectomy, pyeloplasty, pyloromyotomy, spinal arthrodesis for scoliosis, and facial reconstruction for cleft lip/palate (i.e. repair/reconstruction of unilateral/bilateral cleft lip/nasal deformity or palatoplasty). International Classification of Diseases, Ninth Revision (ICD-9) diagnosis and procedure codes and Current Procedural Terminology (CPT) codes were used to identify patients who underwent the above procedures (Additional file 1).

Demographic data included patient age, sex, and race (Asian, African American, white, other, and unknown). For dependents in the MDR with missing race data, race was assigned using the corresponding sponsor’s value [10]. Outcomes included length of hospital stay and all-cause mortality occurring during the hospitalization or follow-up. Follow-up extended 30 days post-operatively for NSQIP-P and 30 days post-discharge, 90 days post-discharge, and beyond (until last follow-up) in the MDR.

For each of the five aforementioned operations, we tabulated demographics and outcomes separately for each data source. Missing data were handled using a complete case approach. We did not conduct statistical tests or provide *p*-values comparing the two large databases, because the goal of the study was to describe the resources rather than to falsify a certain hypothesis test. All analyses were performed using SAS v9.3 (SAS Institute, Inc., Cary, NC). This research was considered exempt by the institutional review boards of the Uniformed Services University of the Health Sciences and Partners Healthcare.

## Results

A total of five procedures were assessed: appendectomy, pyeloplasty, pyloromyotomy, scoliosis operations, and cleft lip/palate repair. Overall, NSQIP-P had a greater number of patients undergoing each procedure. Among both data sources, overall mortality was low, with 1 death reported following 24,965 appendectomies, 9 following 5838 scoliosis operations, and 1 following 6951 cleft lip/palate repairs. There were no mortalities following pyloromyotomy (*n* = 4054) or pyeloplasty (*n* = 898). Results stratified by operation are described in detail below.

### Appendectomy

A total of 20,602 pediatric patients in NSQIP-P and 4363 patients in MDR underwent appendectomy (Table 1). Clinically similar median (IQR) patient ages were observed (NSQIP-P, 11 [8–14] years vs. MDR, 12 [9–15] years). Regarding racial distribution, NSQIP-P included patients who were 2% (*n* = 502) Asian, 9% (*n* = 1783) African American, and 75% (*n* = 15,474) white; the MDR included patients who were 3% (*n* = 139) Asian, 8% (*n* = 345) African American, and 84% (*n* = 3661) white. Median (IQR) length of hospital stay was clinically similar between NSQIP-P (1 [1–3] days) and MDR (0 [0–2] days). There was one (< 0.1%) 30-day mortality recorded in the NSQIP-P and no 30-day mortalities (one 90-day mortality) recorded in the MDR. Proportion of 30-day readmission was slightly higher in NSQIP-P (4%, *n* = 878), compared with MDR (3%, *n* = 126). The 90-day readmission proportion was 3.3% (*n* = 143) in the MDR.

**Table 1:** Appendectomy in NSQIP-P and MDR

Variable	Pediatric NSQIP (*n* = 20,602)	MDR (*n* = 4363)
Female	8154 (39.6)	1779 (40.8)
Age, years	11 [8, 14]	12 [9, 15]
Race		
Asian	502 (2.4)	139 (3.2)
African American	1783 (8.7)	345 (7.9)
Other	139 (0.7)	168 (3.9)
Unknown	2704 (13.1)	50 (1.1)
White	15,474 (75.1)	3661 (83.9)
Year		
2012	5167 (25.1)	1685 (38.7)
2013	7843 (38.1)	1516 (34.8)
2014	7592 (36.9)	1150 (26.4)
Total length of hospital stay, days	1 [1, 3]	1 [0, 2]
Thirty-day mortality	1 (< 0.1)	0 (0.0)
Ninety-day mortality	–	1 (0.0)
Thirty-day readmission	878 (4.3)	126 (2.9)
Ninety-day readmission	–	143 (3.3)

Reported as number (%) or median [interquartile range]. Included years: 2012–2014

### Pyeloplasty

A total of 786 patients in NSQIP-P and 112 in the MDR underwent pyeloplasty (Table 2). Median (IQR) age was slightly greater in MDR, compared with NSQIP (2 [1–6] years vs. 0.9 [0.4–3.4] years). NSQIP-P included patients who were 5% (*n* = 35) Asian, 9% (*n* = 73) African American, and 72% (*n* = 562) white, while the MDR included patients who were 3% (*n* = 3) Asian, 18% (*n* = 16) African American, and 80% (*n* = 90) white. Median (IQR) length of hospital stay was clinically similar between NSQIP-P (1 [1, 2] days) and MDR (0 [0–1] days). There were no mortalities. Proportion of 30-day readmission was similar in both NSQIP-P (7%, *n* = 55) and MDR (7%, *n* = 8). Proportion of 90-day readmissions was 11% (*n* = 12) in the MDR.

**Table 2:** Pyeloplasty in NSQIP-P and MDR

Variable	Pediatric NSQIP (*n* = 786)	MDR (*n* = 112)
Female	220 (28.0)	31 (27.7)
Age, years	0.9 [0.4, 3.4]	2 [1, 6]
Race		
Asian	35 (4.5)	3 (2.7)
African American	73 (9.3)	18 (16.1)
Other	2 (0.3)	1 (0.9)
Unknown	114 (14.5)	0 (0.0)
White	562 (71.5)	90 (80.4)
Year		
2012	206 (26.2)	36 (32.1)
2013	231 (29.4)	49 (43.8)
2014	349 (44.4)	27 (24.1)
Total length of hospital stay, days	1 [1, 2]	0 [0, 1]
Thirty-day mortality	0 (< 0.1)	0 (0.0)
Ninety-day mortality	–	0 (0.0)
Thirty-day readmission	55 (7.0)	8 (7.1)
Ninety-day readmission	–	12 (10.7)

Reported as number (%) or median [interquartile range]. Included years: 2012–2014

### Pyloromyotomy

A total of 3827 patients in NSQIP-P and 227 in the MDR underwent pyloromyotomy (Table 3). Median (IQR) patient age was 34 (27–46) days in NSQIP-P and < 1 year (age recorded in years) in the MDR. NSQIP-P included patients who were 1% (*n* = 42) Asian, 9% (*n* = 345) African American, and 76% (*n* = 2903) white; the MDR included patients who were 2% (*n* = 4) Asian, 8% (*n* = 19) African American, and 87% (*n* = 197) white. Median (IQR) length of hospital stay was clinically similar between NSQIP-P (2 [1–3] days) and MDR (2 [1, 2] days). There was one mortality in the NSQIP-P and none in the MDR. Proportion of 30-day readmission was higher in NSQIP-P (3%, *n* = 118) compared with MDR (1%, *n* = 3). The 90-day readmission proportion was 2% (*n* = 5) in the MDR.

**Table 3:** Pyloromyotomy in NSQIP-P and MDR

Variable	Pediatric NSQIP (*n* = 3827)	MDR (*n* = 227)
Female	647 (16.9)	37 (16.3)
Age, days	34 [27, 46]	--*
Race		
Asian	42 (1.1)	4 (1.8)
African American	345 (9.0)	19 (8.4)
Other	14 (0.4)	3 (1.3)
Unknown	523 (13.7)	4 (1.8)
White	2903 (75.9)	197 (86.8)
Year		
2012	1326 (34.6)	109 (48.0)
2013	1284 (33.6)	63 (27.8)
2014	1217 (31.8)	55 (24.2)
Total length of hospital stay, days	2 [1, 3]	2 [1, 2]
Thirty-day mortality	1 (< 0.1)	0 (0.0)
Ninety-day mortality	–	0 (0.0)
Thirty-day readmission	118 (3.1)	3 (1.3)
Ninety-day readmission	–	5 (2.2)

Reported as number (%) or median [interquartile range]. Included years: 2012–2014

* < 1 year of age (age recorded in years)

### Scoliosis operations

A total of 5743 patients in NSQIP-P and 95 in the MDR underwent arthrodesis for scoliosis (Table 4). A larger majority of patients were female in NSQIP-P (75%, *n* = 4277), compared with MDR (67%, *n* = 64). Patient race in the NSQIP-P was 2% (*n* = 109) Asian, 16% (*n* = 925) African American, and 72% (*n* = 4140) white; the MDR included patients who were 3% (*n* = 3) Asian, 19% (*n* = 18) African American, and 75% (*n* = 71) white. The median (IQR) length of stay was 5 (4–6) days in NSQIP-P vs. 4 (0–6) days in MDR. There were 9 (0.2%) mortalities in NSQIP-P and none in the MDR. The rate of 30-day readmission was lower in NSQIP-P (4%, *n* = 206), compared with MDR (6%, *n* = 6). The 90-day readmission proportion was 11% (*n* = 10) in the MDR.

**Table 4:** Arthrodesis for scoliosis in NSQIP-P and MDR

Variable	Pediatric NSQIP (*n* = 5743)	MDR (*n* = 95)
Female	4277 (74.5)	64 (67.4)
Age, years	14 [13, 16]	14 [12, 16]
Race		
Asian	109 (1.9)	3 (3.2)
African American	925 (16.1)	18 (18.9)
Other	25 (0.4)	2 (2.1)
Unknown	544 (9.5)	1 (1.1)
White	4140 (72.1)	71 (74.7)
Year		
2012	1524 (26.5)	34 (35.8)
2013	1883 (32.8)	36 (37.9)
2014	2336 (40.7)	25 (26.3)
Total length of hospital stay, days	5 [4, 6]	4 [0, 6]
Thirty-day mortality	9 (0.2)	0 (0.0)
Ninety-day mortality	–	0 (0.0)
Thirty-day readmission	206 (3.6)	6 (6.3)
Ninety-day readmission	–	10 (10.5)

Reported as number (%) or median (interquartile range). Included years: 2012–2014

### Cleft lip/palate repair

A total of 6202 patients in NSQIP-P and 749 in the MDR underwent cleft lip/palate repair (Table 5). NSQIP-P included patients who were 10% (*n* = 598) Asian, 7% (*n* = 441) African American, and 69% (*n* = 4294) white. MDR included patients who were 7% (*n* = 49) Asian, 6% (*n* = 45) African American, and 84% (*n* = 626) white. Length of hospital stay was similar between NSQIP-P (median 1, IQR 1–2 days) and MDR (median 0, IQR 0–1 day). There were 3 (< 0.1%) 30-day mortalities in the NSQIP-P and none (one 90-day mortality) in the MDR. Thirty-day readmission was similar (3%, *n* = 196 in NSQIP-P vs. 4%, *n* = 28 in MDR). The 90-day readmission proportion was 7% (*n* = 54) in the MDR.

**Table 5:** Cleft lip/palate repair in NSQIP-P and MDR

Variable	Pediatric NSQIP (*n* = 6202)	MDR (*n* = 749)
Female	2612 (42.1)	337 (45.0)
Age, years	1 [0.5, 2.7]	1 [1, 7]
Race		
Asian	598 (9.6)	49 (6.5)
African American	441 (7.1)	45 (6.0)
Other	67 (1.1)	23 (3.1)
Unknown	802 (12.9)	6 (0.8)
White	4294 (69.2)	626 (83.6)
Year		
2012	1850 (29.8)	292 (39.0)
2013	2172 (35.0)	266 (35.6)
2014	2180 (35.1)	190 (25.4)
Total length of hospital stay, days	1 [1, 2]	0 [0, 1]
Thirty-day mortality	3 (< 0.1)	0 (0.0)
Ninety-day mortality	–	1 (< 0.1)
Thirty-day readmission	196 (3.2)	28 (3.7)
Ninety-day readmission	–	54 (7.2)

Reported as number (%) or median [interquartile range]. Included years: 2012–2014

## Discussion

In this study comparing basic characteristics and outcomes of pediatric patients undergoing common procedures in the NSQIP-P and MDR, we report that the age and sex of patients were overall similar as were mortality, length of hospital stay, and readmission rate. Race distribution for each procedure was similar between the two databases, with the MDR containing a generally slightly higher proportion of white patients, although the distribution varied between procedures. Based on these comparisons, we review the advantages and disadvantages of each database, in order to help guide researchers toward the most suitable resource for a given analysis.

Two important advantages of the MDR relative to NSQIP-P are follow-up duration and patient demographics. First, the MDR does not limit outcomes to 30 days, whereas the NSQIP-P limits follow-up to 30 days post-operatively. For some outcomes, 30 days may be adequate follow-up; for others, it may be advantageous to study longer term endpoints. For example, 90-day readmission rates were often notably higher than 30-day readmission rates in the MDR. Finally, as (non-sampled) claims data, the MDR captures 100% of operations. In contrast, the NSQIP-P intentionally captures only a select number of operations in order to maximize sampling efficiency.

There are also important disadvantages to the MDR, compared with the NSQIP-P. As a clinical database, NSQIP-P captures specific post-operative outcomes, including surgical site infections, need for mechanical ventilation, pneumonia, and others. The NSQIP-P is specifically designed as a quality improvement initiative, whereas the data in the MDR are captured as claims. These outcomes have been validated in the NSQIP and may not be as reliably captured in the MDR, unless a specific reimbursement code was applied to the event. As such, it is important to choose objective outcomes whenever able if the MDR is to be used; whereas, if the question of interest involves clinical endpoints captured by the NSQIP, the latter dataset may be preferable.

Additionally, NSQIP-P tracks infants under 30 days old, whereas the MDR poses a challenge in tracking infants under 1 year of age, due to the lag time in establishing them in the database as new patients with unique identifiers. Furthermore, age is documented in integer years in the MDR, whereas the NSQIP-P records age in days for neonatal patients. Given these differences, for neonatal patients, the NSQIP-P may be a preferable resource.

Finally, although both NSQIP-P and the MDR are updated on a yearly basis, NSQIP-P data are more readily accessible by the medical community, being provided at no charge to researchers and participating hospitals. In contrast, access to the MDR is free but controlled by Federal oversight and requires a more extensive application process.

This study has several strengths, such as the inclusion of surgical procedures with markedly different rates of use and occurring in different body systems. Both databases draw from sufficiently large populations so as to capture infrequent procedures, and both have demonstrated suitability as tools for health services research. Therefore, comparing the MDR and the NSQIP-P provides useful information on the scope of these databases as well as the difference between them.

There are also important limitations to consider. As described above, the MDR is a claims database which is limited in its capture of outcomes, and may not address subtler clinical findings. Similarly, the number of operations captured differs between the databases with NSQIP containing many-fold more operations than the MDR. This may occur because of the different inclusion criteria for each database. Specifically, the NSQIP systematically samples patients who undergo selected operations from participating institutions. Conversely, the MDR contains a large cohort of children insured via TRICARE, and can be interrogated using claims codes to identify all patients who have undergone a given operation over time. Finally, this study assessed only a few notable outcomes (demographics, length of stay, mortality, and readmissions), which together serve as a strong foundation for comparison of the two databases, but which cannot constitute a full validation. Further research is needed to address each of these issues.

## Conclusions

In conclusion, the MDR was found to be comparable to the NSQIP-P in several areas including patient demographics and several clinical outcomes following five common pediatric surgical operations. Additional comparison to other standard databases with other populations will further establish the MDR as a tool for robust health services research relevant to the general United States population.

## Additional file

**Additional file 1: Table S1.** ICD-9 and CPT codes for included procedures and diagnoses.

## Data Availability

Access to the MDR database is free but controlled by Federal oversight. Access to the NSQIP-P database is free for participating hospitals.

## Abbreviations

CPT: Current Procedural Terminology
ICD-9: International Classification of Diseases, Ninth Revision
IQR: Interquartile range
LOS: Length of stay
MDR: Military Health System Data Repository
MHS: Military Health System
NSQIP-P: National Surgical Quality Improvement Program-Pediatric
