# Supplementary material for: Comparison of Military Health System Data Repository and American College of Surgeons National Surgical Quality Improvement Program-Pediatric
Source: BMC Pediatr. 2019 Nov 8;19:419. doi: 10.1186/s12887-019-1795-x (PMC6839070; doi:10.1186/s12887-019-1795-x)
Supplement: Supplementary file 1 — Additional file 1: Table S1. ICD-9 and CPT codes for included procedures and diagnoses. [file 12887_2019_1795_MOESM1_ESM.docx]

**Table S1**. ICD-9 and CPT codes for included procedures and diagnoses.

| ***Operation*** | ***Diagnosis*** | ***ICD-9 diagnosis code*** | ***ICD-9 procedure code*** | ***CPT code*** |
| --- | --- | --- | --- | --- |
| Appendectomy | Acute appendicitis | 540x | 47.0, 47.01, 47.09 | 44950, 44960, 44970, 44979 |
| Pyeloplasty | Ureteropelvic junction obstruction | 753.21 | 55.87 | 50400, 50405, 50544 |
| Pyloromyotomy | Pyloric stenosis | 750.5 | 43.3 | 43520, 43659 |
| Arthrodesis | Scoliosis | 737.30-737.34, 737.39 | 81.00-81.08 | 22800, 22802, 22804, 22808, 22810, 22812 |
| Cleft lip/palate repair | Cleft lip/palate | 749.00-749.04, 749.10-749.14, 749.20-749.25 | 27.54, 27.57, 27.59, 27.62, 27.69 | 40700-40702, 42200, 42205, 42210, 42225-6, 42235 |

*ICD-9*, International Classification of Diseases, Ninth Revision (ICD-9); *CPT*, Current Procedural Terminology
